# Push–pull fluorophores based on NHS esters of bithiophene for labelling of biomolecules containing primary amines

**DOI:** 10.1098/rsos.241816

**Published:** 2025-02-26

**Authors:** Mariana Barros, Pau Arroyo, Jose A. Sáez, Salvador Gil, Margarita Parra, Susana P. G. Costa, M. Manuela M. Raposo, Pablo Gaviña

**Affiliations:** ^1^ Instituto Interuniversitario de Investigación de Reconocimiento Molecular y Desarrollo Tecnológico (IDM), Universitat de València, Universitat Politècnica de València, c/ Doctor Moliner 50, Burjassot, Valencia 46100, Spain; ^2^ Departamento de Química Orgánica, Universitat de València, c/ Doctor Moliner 50, Burjassot, Valencia 46100, Spain; ^3^ CIBER de Bioingeniería, Biomateriales y Nanomedicina (CIBER-BBN), Madrid, Spain; ^4^ Center of Chemistry, University of Minho, Campus de Gualtar, Braga 4710-57, Portugal

**Keywords:** fluorescent labelling, push–pull heterocyclic fluorophores, bithiophene, Boc-lysine, primary amine chemosensor

## Abstract

Fluorescent labelling is a versatile tool to visualize biomolecules containing primary amines in their cellular environment, allowing the study of their function or interactions. Here, three organic fluorophores that can irreversibly bind to the primary amine group on the target biomolecule are reported. They consist of push–pull heterocyclic dyes based on bithiophene and incorporating a terminal *N*-hydroxysuccinimidyl ester as a reactive group for labelling primary amine groups from biomolecules as (poly)amines, peptides or proteins. Their potential as chemosensors for primary amines, using N_α_-Boc protected amino acid l-lysine as a model, was assessed through UV–Visible, fluorescence and ^1^H NMR titrations.

## 1. Introduction

Biomolecules containing primary amines, such as (poly)amines, peptides and proteins, are essential for nearly every cellular function, and fluorescence-based imaging techniques provide powerful tools for investigating their activity in real time within living cells [1,2]. Such capabilities are crucial for advancing our understanding of cellular processes.

Over the past several decades, numerous fluorescent chemosensors have been developed, becoming indispensable tools for bioimaging and disease investigation [3]. Small-molecule fluorescent compounds are essential as labelling agents and activatable sensors because they are versatile, cost-effective, easy to handle and can achieve high signal-to-noise ratios through advanced chemical design [4]. Current fluorescence-based imaging technology heavily relies on small molecule-based dyes owing to their compact size, facile chemical modification, excellent reproducibility and biocompatibility [5]. Among the developed organic small molecule-based fluorescent dyes for biomolecule labelling are rhodamine [6], coumarin [7], fluorescein [8], BODIPY [9] and cyanine [10].

The abundance of reactive biogenic primary amines, i.e. the N-terminus and lysine residues, on protein surfaces allows for easy labelling of any protein with amine-reactive chemical groups [11]. The same principle can be applied to any biomolecule containing primary amine functional groups. It is well established that *N*-hydroxysuccinimidyl (NHS) esters can react with primary amino groups under physiological conditions. This reaction is highly efficient and specific, making it a preferred method for the labelling of biomolecules containing primary amines. By conjugating fluorescent dyes to NHS esters, it is possible to directly attach these dyes to biomolecules containing primary amines, allowing their visualization under fluorescence microscopy [12–14].

**Figure 1.**
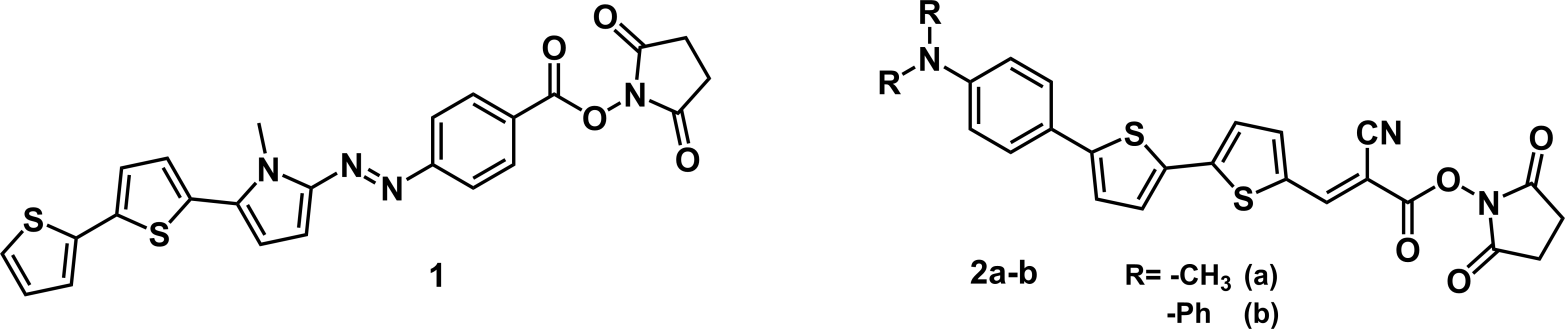
Structure of the prepared bithiophene-based push–pull fluorophores.

Based on our previous experience in the synthesis of push–pull heterocyclic dyes based on pyrrole and bithiophene [15–20], we decided to prepare several bithiophene-based push–pull fluorophores modified with an NHS ester as a reactive group (figure 1) to form an amide bond with the primary amino groups of biomolecules. Using Boc-lysine as a model system, we demonstrate the effectiveness and use of this labelling technique. To the best of our knowledge, this is the first example of the use of bithiophene-based push–pull fluorophores for the labelling of molecules via NHS esters.

## 2. Material and methods

### 2.1. General methods

The reagents and solvents used in the syntheses were purchased from Sigma-Aldrich and were used without further purification. Proton and ^13^C nuclear magnetic resonance (^1^H NMR and ^13^C NMR) spectra were recorded on Bruker Avance 300, 400 or 500 MHz equipment and referenced to the solvent peak at 25°C. The solvents are indicated in parentheses before the chemical shift values (δ relative to tetramethyl silane (TMS) and given in ppm). High-resolution mass spectra (HRMS) were recorded in the positive ion mode on an AB SCIEX Triple TOF^TM^ 5600 liquid chromatography/mass spectrometry spectrometer. Fluorescence measures were taken with a Shimadzu RF-6000 spectrofluorophotometer using a 1 cm path length cuvette. UV–Visible (UV-Vis) absorption spectra were registered with a Shimadzu UV-2600 spectrophotometer, using a 1 cm path length cuvette.

### 2.2. Syntheses

#### 2.2.1. Synthesis of bithienyl-pyrrole **5**

Precursor **5** was synthesized through a Suzuki coupling reaction following a reported procedure [21].

2-Bromo-2,2′-bithiophene (**3**) (1 equiv.) was coupled with 1-methyl-1*H*-pyrrole-2-boronic acid pinacol ester **4** (1.2 equiv.) in a mixture of dimethoxyethane (DME, 10 ml), aqueous 2 M Na_2_CO_3_ (1 ml) and Pd(PPh_3_)_4_ (5 mmol%) at 80°C under nitrogen atmosphere. The reaction was monitored by thin-layer chromatography (TLC), which determined the reaction time (16 h). After cooling, the mixture was extracted with AcOEt (20 ml) and H_2_O (3 × 20 ml), and the phases were separated. The organic phase obtained was dried over anhydrous MgSO_4_, filtered and the solvent was evaporated under reduced pressure to give a crude mixture. The crude product was purified through a silica gel chromatography column using mixtures of dichloromethane and light petroleum of increasing polarity to afford product **5** as a dark green oil (131 mg, 66% yield). ^1^H NMR (400 MHz, CDCl_3_) δ 7.21 (*dd*, *J* = 5.1, 1.2, 1H), 7.17 (*dd*, *J* = 3.6, 1.2, 1H), 7.12 (*d*, *J* = 3.7, 1H), 7.04–6.99 (*m*, 2H), 6.92 (*d*, *J* = 3.7, 1H), 6.71 (*t*, *J* = 2.3, 1H), 6.35 (*dd*, *J* = 3.7, 1.4, 1H), 3.76 (*s*, 3H) ppm.

#### 2.2.2. Synthesis of **7**

Compound **7** was obtained through an azo coupling as previously reported [16].

Hydrochloric acid 37% (333 μl) was added to a round-bottom flask with a suspension of 4-aminobenzoic acid (0.067 g, 1.0 equiv.) in water (833 μl) and stirred at 0–5°C until the mixture was homogenous. NaNO_2_ (0.041 g, 1.2 equiv.) dissolved in water (666 μl) was slowly added to the well-stirred previous solution. The reaction mixture was stirred for 30 min at 0–5°C. A solution with **5** (0.120 g, 1 equiv.) was prepared in MeOH (3.333 ml) and pyridine (167 μl). The previously prepared diazonium salt solution was added dropwise to the solution of **5** at 0–5°C. The resulting mixture was stirred for 3 h at 0–5°C and then concentrated under reduced pressure. The precipitate was filtered, washed with cold water and dried to afford the pure heterocyclic azo derivative **7** as a grey solid (164 mg, 83% yield). ^1^H NMR (400 MHz, deuterated dimethyl sulfoxide (DMSO-*d*_6_)) δ 8.06 (*d*, *J* = 8.5, 2H), 7.86 (*d*, *J* = 8.6, 2H), 7.59 (*dd*, *J* = 5.1, 1.2, 1H), 7.57 (*d*, *J* = 3.9, 1H), 7.45 (*d*, *J* = 3.9, 1H), 7.43 (*dd*, *J* = 3.6, 1.2, 1H), 7.14 (*dd*, *J* = 5.1, 3.6, 1H), 6.95 (*d*, *J* = 4.5, 1H), 6.84 (*d*, *J* = 4.5, 1H), 4.13 (*s*, 3H) ppm.

#### 2.2.3 General procedure for the synthesis of aldehydes **12a–b**

Aldehydes **12a–b** were prepared by Suzuki coupling as previously described [21].

5′-Bromo-2,2′-bithiophene-5-carboxaldehyde **11** (0.73 mmol) was coupled with boronic acids **9** and **10** (0.88 mmol) in a mixture of DME (10 ml), aqueous 2 M Na_2_CO_3_ (1 ml) and Pd(PPh_3_)_4_ (5 mol%) at 80°C under nitrogen atmosphere. The reactions were monitored by TLC, which determined the different reaction times (17–21 h). After cooling, the mixture was filtered and washed with water and diethyl ether to afford aldehydes **12a–b**.

**12a** as an orange solid (213 mg, 93% yield) ^1^H NMR (400 MHz, CDCl_3_) δ 9.84 (*s*, 1H), 7.66 (*d*, *J* = 4.0, 1H), 7.53–7.47 (*m*, 2H), 7.30 (*d*, *J* = 3.9, 1H), 7.21 (*d*, *J* = 4.0, 1H), 7.11 (*d*, *J* = 3.9, 1H), 6.76–6.70 (*m*, 2H), 3.01 (*s*, 6H) ppm. HRMS: *m*/*z* calcd for C_17_H_16_NOS_2_ [M+H]^+^: 314.0673; found: 314.0675.

**12b** as an orange solid (172 mg, 54% yield) ^1^H NMR (400 MHz, acetone-*d*_6_) δ 9.92 (*s*, 1H), 7.93 (*d*, *J* = 4.0, 1H), 7.66−7.58 (*m*, 2H), 7.52 (*d*, *J* = 3.9, 1H), 7.47 (*d*, *J* = 4.0, 1H), 7.42 (*d*, *J* = 3.9, 1H), 7.40−7.30 (*m*, 4H), 7.16−7.07 (*m*, 6H), 7.09−7.01 (*m*, 2H) ppm. HRMS: *m*/*z* calcd for C_27_H_19_NOS_2_ [M]^+^: 437.0908; found: 437.0891.

#### 2.2.4 General procedure for the synthesis of derivatives **14a–b**

Cyanoacetic derivatives **14a–b** were synthesized by a Knoevenagel condensation with cyanoacetic acid as previously described [16].

To a mixture of aldehyde **12** (1 equiv.) and cyanoacetic acid (**13**) (1.2 equiv.) in acetonitrile (ACN, 10 ml) was added one drop of piperidine. The reaction mixture was heated under reflux for 3−4 h and then cooled to 0°C. The precipitates obtained were filtered and washed with ethyl ether to give the pure products **14a–b**.

**14a** as a red solid (120 mg, 63% yield) ^1^H NMR (400 MHz, DMSO-*d*_6_) δ 8.10 (*s*, 1H), 7.69 (*d*, *J* = 3.9, 1H), 7.52 (*d*, *J* = 8.9, 2H), 7.44 (*d*, *J* = 3.9, 1H), 7.41 (*d*, *J* = 3.9, 1H), 7.32 (*d*, *J* = 3.9, 1H), 6.75 (*d*, *J* = 8.9, 2H), 2.95 (*s*, 6H) ppm.

**14b** as a red solid (132 mg, 96% yield) ^1^H NMR (400 MHz, DMSO-*d*_6_) δ 8.03 (*s*, 1H), 7.68−7.58 (*m*, 3H), 7.50−7.40 (*m*, 3H), 7.34 (*t*, *J* = 7.9, 4H), 7.15−7.03 (*m*, 6H), 7.00−6.94 (*m*, 2H) ppm.

#### 2.2.5 Synthesis of dyes **1** and **2a–b**

In a typical procedure, a mixture of **7** or **14a–b** (1 equiv.), NHS (5 equiv.) and *N*-(3-dimethylaminopropyl)-*N'*-ethylcarbodiimide hydrochloride (EDC·HCl, 5 equiv.) in dichloromethane (DCM, 25 ml) was stirred at room temperature for 24 h. The mixture was extracted with H_2_O (3 × 10 ml), and the phases were separated. The organic phase obtained was dried over anhydrous MgSO_4_, filtered and the solvent was evaporated under reduced pressure. The pure dye was obtained by recrystalization from DCM/hexane.

Dye **1** as a dark red solid (125 mg, 69% yield) ^1^H NMR (500 MHz, DMSO-*d*_6_) δ 8.21 (*d*, *J* = 8.7, 2H), 7.99 (*d*, *J* = 8.7, 2H), 7.62−7.56 (*m*, 2H), 7.48−7.41 (*m*, 2H), 7.15 (*dd*, *J* = 5.1, 3.6 Hz, 1H), 6.94 (*d*, *J* = 4.5 Hz, 1H), 6.85 (*d*, *J* = 4.5 Hz, 1H), 4.16 (*s*, 3H), 2.91 (*s*, 4H) ppm. ^13^C NMR (125 MHz, DMSO-*d*_6_) δ 170.85, 131.95, 131.40, 129.08, 128.83, 126.75, 125.63, 125.28, 122.77, 113.98, 32.08, 26.03 ppm. HRMS: *m*/*z* calculated for C_24_H_18_N_4_O_4_S_2_ [M+H]^+^: 491.0848; found 491.0831.

Dye **2a** as a burgundy solid (52 mg, 34% yield). ^1^H NMR (500 MHz, DMSO-*d*_6_) δ 8.79 (*s*, 1H), 8.18 (*d*, *J* = 4.3 Hz, 1H), 7.70 – 7.42 (*m*, 5H), 6.75 (*d*, *J* = 8.3 Hz, 2H), 2.97 (*s*, 6H), 2.88 (*s*, 4H) ppm. ^13^C NMR (125 MHz, DMSO-*d*_6_) δ 173.24, 170.50, 159.93, 151.16, 151.06, 149.31, 146.04, 133.33, 131.41, 130.63, 127.20, 125.36, 123.40, 120.64, 115.61, 112.74, 88.44, 25.99, 25.69 ppm. HRMS: *m*/*z* calculated for C_24_H_19_N_3_O_4_S_2_ [M+H]^+^: 478.0897; found 478.0864.

Dye **2b** as a dark red solid (134 mg, 86% yield). ^1^H NMR (500 MHz, DMSO-*d*_6_) δ 8.82 (*s*, 1H), 8.19 (*d*, *J* = 4.3 Hz, 1H), 7.74 (*d*, *J* = 4.0 Hz, 1H), 7.69 (*d*, *J* = 4.2 Hz, 1H), 7.63 (*dd*, *J* = 9.9, 3.2 Hz, 2H), 7.52 (*d*, *J* = 4.0 Hz, 1H), 7.34 (*ddd*, *J* = 11.4, 7.4, 2.9 Hz, 4H), 7.13–7.04 (*m*, 6H), 6.95 (*dd*, *J* = 8.9, 2.4 Hz, 2H), 2.88 (*s*, 3H) ppm. ^13^C NMR (125 MHz, DMSO-*d*_6_) δ 173.23, 170.46, 159.80, 152.17, 151.24, 150.44, 147.03, 133.82, 133.09, 130.19, 127.29, 126.45, 125.84, 125.25, 124.36, 122.61, 89.23, 25.72 ppm. HRMS: *m*/*z* calculated for C_34_H_23_N_3_O_4_S_2_ [M+H]^+^: 602.1208; found 602.1173.

### 2.3. UV–Visible and fluorescence titrations with N_α_-Boc protected amino acid l-lysine

In a 3 ml quartz cell (1.0 cm of path length), to 2800 μl of a 10 μM solution of the corresponding dye (**1**, **2a** or **2b**) in DMSO/H_2_O 99 : 1, increasing additions of 10 μl of N_α_-Boc protected amino acid l-lysine (Boc-Lys-OH; 3 mM in aq NaHCO_3_ 10 mM buffer, pH 8.4) were added. For each aliquot added, the solution was stirred for 5 s and then the corresponding UV–Vis absorption and fluorescence emission spectra (at the corresponding excitation wavelength) of the solution were recorded.

### 2.4. ^1^H NMR titrations

A known amount of the corresponding dye was dissolved in DMSO-*d*_6_ (*ca* 400 μl), in an NMR tube in such a way that the final concentration of the dye was 10 mM. Then, increasing amounts of Boc-Lys-OH in MeOD-*d*_4_ (10 μl aliquots, 0−4 equiv.) were added to the tubes, and finally, more DMSO-*d*_6_ was added to obtain a total volume of 500 μl. The solutions were shaken for 5 min at room temperature, and the corresponding ^1^H NMR spectra were recorded.

## 3. Results and discussion

### 3.1. Synthesis of dye **1**

**Scheme 1.**
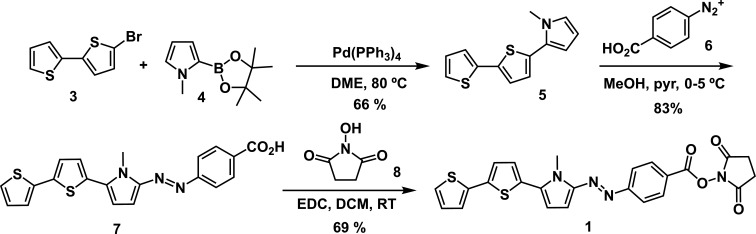
Synthetic pathway for the preparation of dye **1**.

The first prepared dye based on bithiophene (compound **1**) was the NHS ester derivative of the azo compound **7** (scheme 1). Compound **7** contains an electron-rich π-bridge/donating moiety on one side of the azo group and an electron-withdrawing benzoic acid group on the other side, which will be used later to bind NHS. On the other hand, the bithienylpyrrole precursor **5** was synthesized in 66% yield through a Suzuki cross-coupling reaction between the commercially available 5-bromo-2,2′-bithiophene **3** and 1-methyl-1*H*-pyrrol-2-yl-2-boronic acid **4** in DME under an inert atmosphere [21]. Diazotation of 4-aminobenzoic acid with NaNO_2_ in HCl at 0−5°C afforded the corresponding diazonium salt **6**, which was further reacted with bithienylpyrrole 5 in methanol and pyridine at 0−5°C to give the heterocyclic azo dye **7,** which was isolated by precipitation as a grey solid in 83% yield. The terminal carboxyl group of the azo derivative was transformed into the NHS ester by using NHS with 1-ethyl-3-(3-dimethylaminopropyl)carbodiimide (EDC) as a coupling agent. The final new product was obtained with a 69% yield after recrystallization.

### 3.2. Synthesis of dyes **2a–b**

**Scheme 2.**
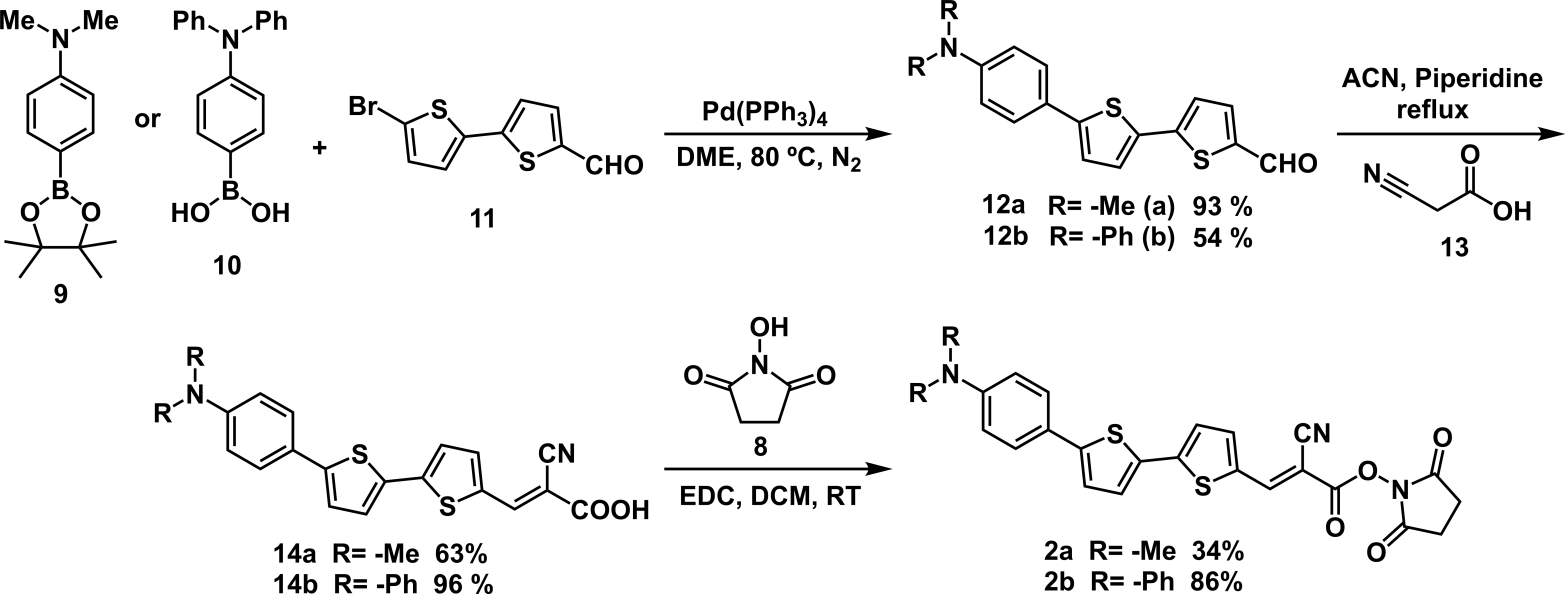
Synthetic pathway for the preparation of dyes **2a–b**.

Another family of bithiophene-containing push–pull dyes was synthesized (scheme 2). They consist of two push–pull molecular systems, containing an *N*,*N*-dialkylaniline as the donor moiety, connected through a bithiophene π-bridge to a cyanoacetic acid derivative as the electron acceptor moiety. The carboxyl group of the cyanoacetic acid was later transformed into an NHS ester as an anchoring group. Formyl compounds **12a–b** incorporating a dialkylaniline group connected to the 2,2-bithiophene moiety were synthesized through a Suzuki cross-coupling reaction of commercially available aryl-boronic acids **9** and **10** with 5′-bromo-5-formyl-2,2′-bithiophene **11** in DME and aqueous 2 M Na_2_CO_3_, using Pd(PPh_3_)_4_ as a palladium catalyst, under an inert atmosphere. In this case, products **12a** and **12b** were obtained with respective yields of 93 and 54%, which may be understandable owing to the different electron-donating character of the dialkyl or diaryl substituent. These formyl precursors were converted into the correspondent cyanoacetic acid derivatives **14a–b** by Knoevenagel condensation with 2-cyanoacetic acid in refluxing ACN in the presence of piperidine as a catalyst, in standard conditions as previously described [16] and with good yields (63 and 96%, respectively). The carboxyl group was then transformed into the NHS ester using NHS with EDC as a coupling agent. The final products were obtained with 34% (**2a**) and 86% (**2b**) yield, respectively.

The new dyes **1** and **2a–b** were fully characterized using ^1^H and ^13^C NMR and HRMS.

### 3.3. Preliminary sensing experiments with N_α_-Boc protected amino acid l-lysine

To evaluate the labelling of biomolecules containing primary amine groups through dyes **1** and **2a–b**, a preliminary study was undertaken using Boc-Lys-OH as a model. The selection of this N-terminal protected amino acid with a side chain containing a primary amine group as a target molecule allowed for evaluating the capabilities of the synthesized dyes as chemical sensors.

**Figure 2.**
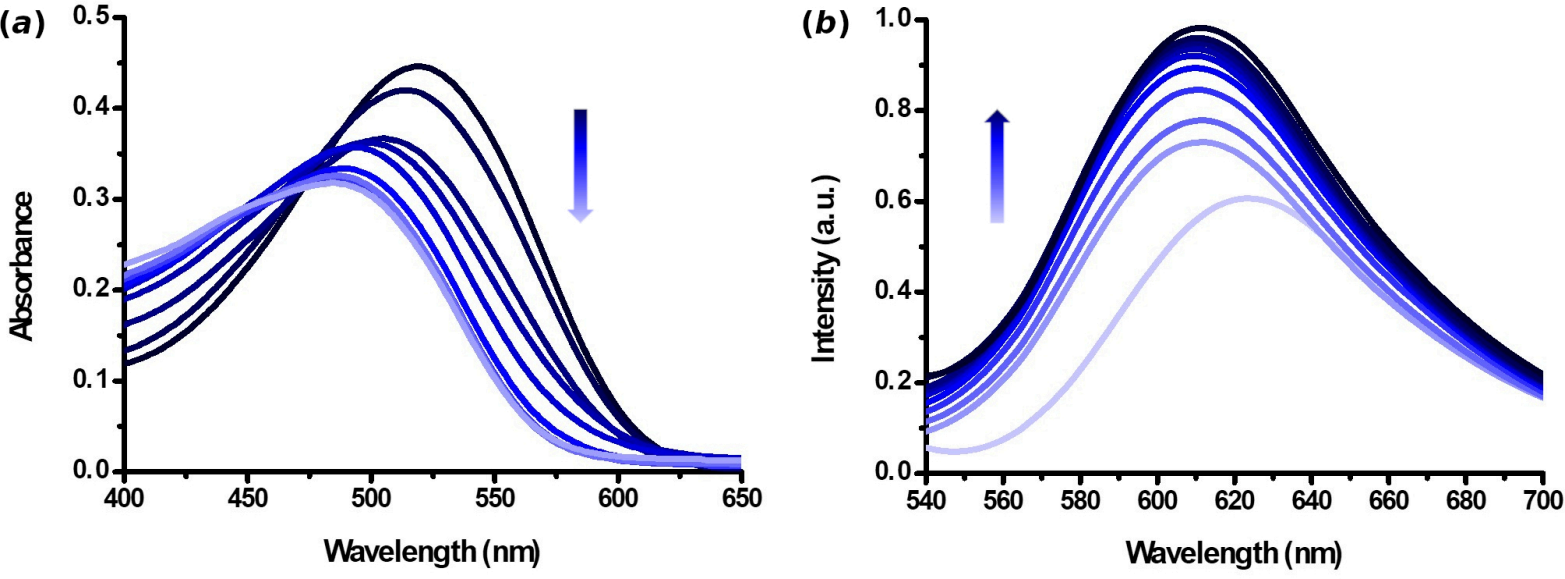
(*a*) UV–Vis and (*b*) fluorescence titration of dye **1** (10 µM in DMSO) with Boc-Lys-OH (0−20 equiv.) in NaHCO_3_ buffer (pH = 8.4).

**Figure 3.**
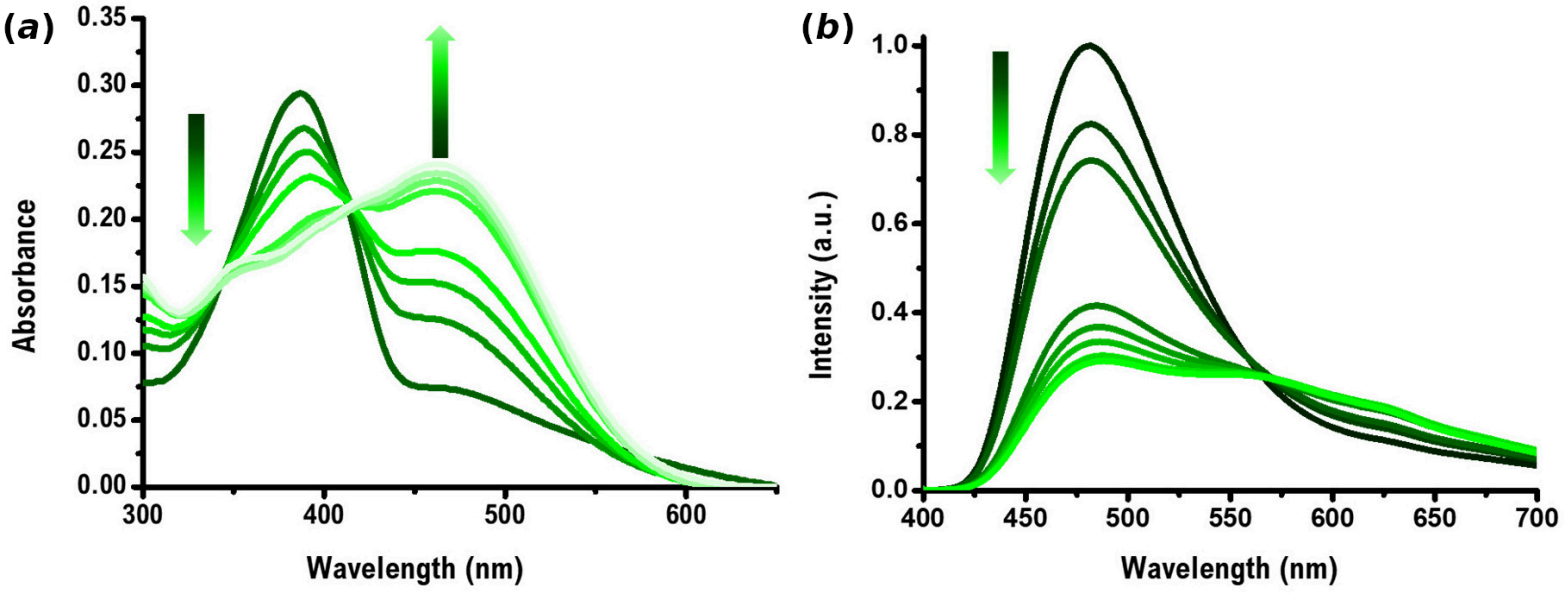
(*a*) UV–Vis and (*b*) fluorescence titration of dye **2** a (10 µM in DMSO) with Boc-Lys-OH (0−15 equiv.) in NaHCO_3_ buffer (pH = 8.4).

For this purpose, UV–Vis and fluorescence titration studies with dyes **1** and **2a–b** in the presence of increasing amounts of Boc-Lys-OH were performed. In a standard procedure, DMSO solutions of dyes **1** and **2a–b** (10 µM) were treated with increasing amounts of Boc-Lys-OH in NaHCO_3_ buffer pH = 8.4 (10 mM), and the corresponding UV–Vis and fluorescence spectra were recorded (figures 2 and 3; electronic supplementary material, figure S11).

Dye **1** (10 µM in DMSO) presents an absorption maximum at 519 nm and an emission maximum at 623 nm when excited at the absorption maximum. The addition of Boc-Lys-OH leads to hypochromic and hypsochromic shifts with a new maximum at 484 nm in the UV–Vis spectra (figure 2*a*). An enhancement of fluorescence emission was observed at 611 nm in the presence of an excess of Boc-Lys-OH (figure 2*b*). Dye **2** a (10 µM in DMSO, NaHCO_3_ buffer, pH = 8.4) exhibits an absorption maximum at 387 nm. Upon adding increasing amounts of Boc-Lys-OH, the absorbance decreases in the peak at 387 nm, and a new maximum peak emerges at 464 nm. Fluorescence titration studies (*λ*_exc_ = 387 nm) were also carried out (figure 3*b*). In the absence of Boc-Lys-OH, compound **2a** showed an emission maximum at 481 nm, which was quenched by the addition of increasing amounts of the amino acid.

Dye **2b** (10 µM in DMSO) shows a maximum absorption at 439 nm and an emission maximum at 621 nm when excited at the absorption maximum (see the electronic supplementary material, figure S11). Of the synthesized dyes, dye **2b** showed the smallest variations in its UV–Vis and fluorescence spectra in the presence of Boc-Lys-OH, indicating a less pronounced response compared to the other dyes studied.

To test the dyes in a more realistic biological environment, fluorescence titration experiments of dye **2a** with BOC-Lys-OH were conducted in a 1 : 1 mixture of DMSO and Roswell Park Memorial Institute (RPMI) 1640 medium (a cell culture medium with l-glutamine and sodium bicarbonate). As observed in the electronic supplementary material, figure S14, the fluorescence emission maximum of **2a**, which appears at 511 nm in the absence of Boc-Lys-OH, experiences a gradual quenching upon increasing additions of the amino acid, similar to that observed in DMSO.

**Figure 4:**
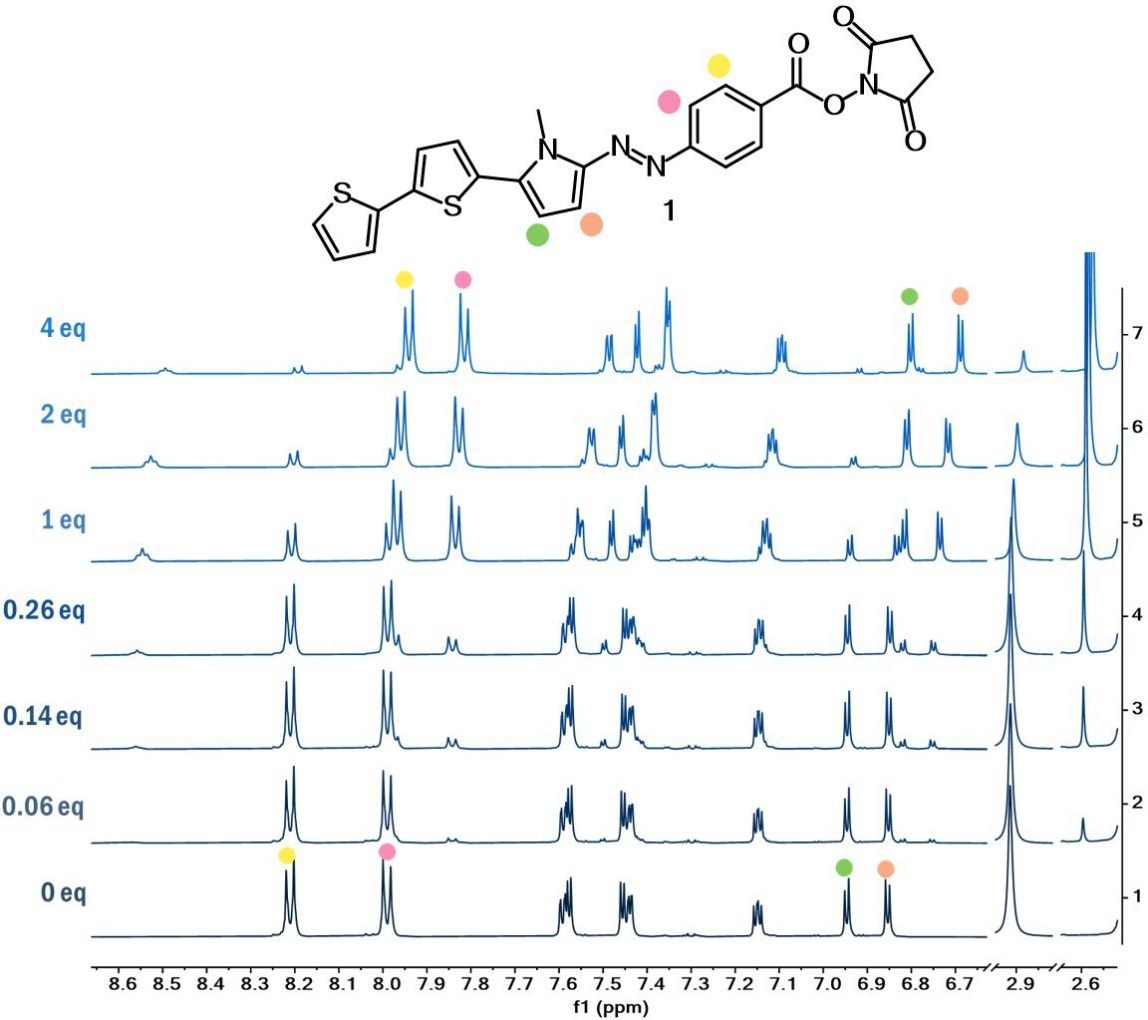
.^1^H NMR studies of dye **1** in DMSO-*d_6_* with increasing amounts of Boc-Lys-OH.

**Figure 5:**
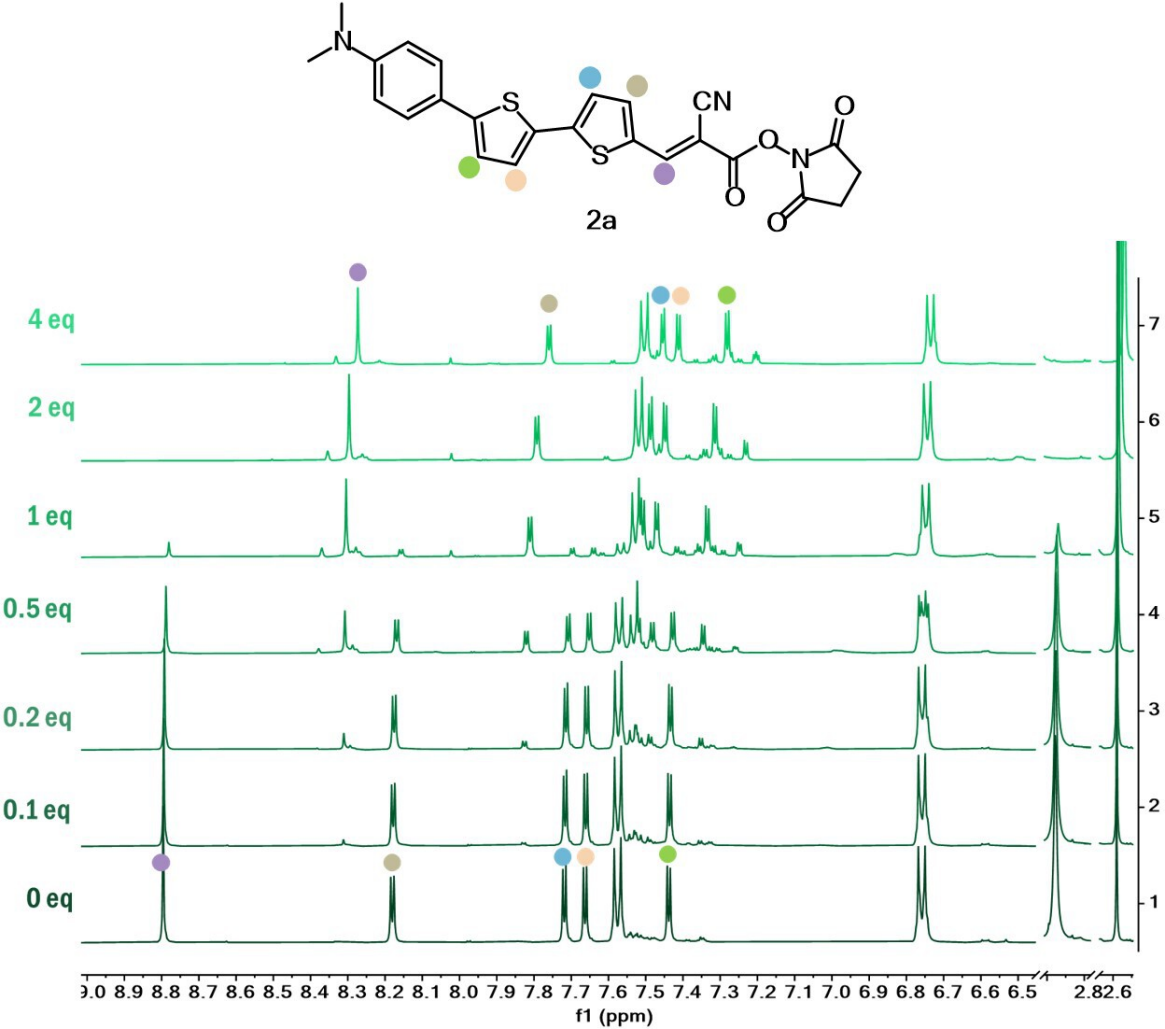
.^1^H NMR studies of dye **2** a in DMSO-*d*_6_ with increasing amounts of Boc-Lys-OH.

Finally, ^1^H NMR investigations were also conducted with dyes **1** and **2a–b**, using increasing amounts of Boc-Lys-OH (figures 4, 5; electronic supplementary material, figure S12). Dye **2b** presented the same trend as for dye **2a**. As anticipated, the formation of the amide bond resulted in an upfield chemical shift of the hydrogens close to the carbonyl moiety, especially of the aromatic protons on the benzene ring of dye **1** and the olefinic hydrogen of compounds **2a–b**. Furthermore, it was noted that the protons associated with the NHS in ester form at 2.9 ppm disappeared, revealing a new singlet at 2.6 ppm characteristic of the free NHS.

## 4. Conclusions

Three push–pull heterocyclic dyes based on bithiophene, incorporating a terminal NHS ester as an anchoring group for irreversibly binding to an amino group of a biomolecule through the formation of an amide bond, were successfully synthesized and characterized. Their potential as chemosensors for biomolecules containing primary amines, using Boc-Lys-OH as a model amino acid incorporating a primary amino group in its side chain, was assessed through UV–Vis, fluorescence and ^1^H NMR titrations. UV–Vis and fluorescence titrations of the dyes in the presence of increasing amounts of Boc-Lys-OH showed significant changes in their UV–Vis and fluorescence spectra, particularly for dyes **1** and **2a**. In addition, the potential of dye **2a** as a chemosensor for primary amines was also assessed in a more competitive medium, such as RPMI 1640 cell culture medium, which showed a quenching of the fluorescence emission in the presence of increasing amounts of Boc-Lys-OH. The ^1^H NMR titrations provided valuable insights, allowing us to visualize the formation of the amide bond between the synthesized dyes and Boc-Lys-OH. The distinctive changes in the ^1^H NMR spectra confirmed the successful reaction. Future studies will focus on optimizing these dyes towards their interaction with primary amine-containing proteins and studying their labelling properties using confocal microscopy.

## Data Availability

The data supporting this article have been uploaded as part of the eletronic supplementary material [22].
